# GABAergic and cholinergic modulation of repetition suppression in inferior temporal cortex

**DOI:** 10.1038/s41598-018-31515-1

**Published:** 2018-09-03

**Authors:** Pradeep Kuravi, Rufin Vogels

**Affiliations:** 1 Laboratorium voor Neuro- en Psychofysiologie, Department of Neurosciences, KU Leuven, Leuven, Belgium; 2 Leuven Brain Institute, Leuven, Belgium

## Abstract

Neurons in many brain areas of different species reduce their response when a stimulus is repeated. Such adaptation or repetition suppression is prevalent in inferior temporal (IT) cortex. The mechanisms underlying repetition suppression in IT are still poorly understood. Studies in rodents and *in-vitro* experiments suggest that acetylcholine and GABA can contribute to repetition suppression by interacting with fatigue-related or local adaptation mechanisms. Here, we examined the contribution of cholinergic and GABAergic mechanisms to repetition suppression in macaque IT, using an adaptation paradigm in which familiar images were presented successively with a short interstimulus interval. We found that intracortical local injections of acetylcholine and of the GABA_A_ receptor antagonist Gabazine both increased repetition suppression in awake macaque IT. The increased repetition suppression was observed for both spiking activity and local field potential power. The latter was present mainly for frequencies below 50 Hz, spectral bands that typically do not show consistent repetition suppression in IT. Although increased with drug application, repetition suppression remained stimulus selective. These findings agree with the hypothesis that repetition suppression of IT neurons mainly results from suppressed input from upstream and other IT neurons but depend less on intrinsic neuronal fatigue.

## Introduction

Many brain areas of several species show adaptation: their neural response to a stimulus changes after exposure to that stimulus. Such adaptation-induced response changes can vary from suppression of the response (“repetition suppression”) to enhancement^1^. Repetition suppression is observed in fMRI activations in humans^2,3^ and nonhuman primates^3,4^ and this likely reflects the suppression observed in neural responses^5^. For repeated visual stimuli, suppressed human fMRI responses are commonly but not exclusively observed in ventral visual stream areas. Likewise, macaque inferior temporal (IT) cortical neurons show substantial repetition suppression^5–9^, both for spiking activity and for local field potential (LFP) power in frequency bands typically higher than 50 Hz^10,11^. The mechanisms underlying repetition suppression in ventral visual stream areas are still unclear. Proposed mechanisms range from bottom-up and local fatigue-related adaptation mechanisms, such as post-synaptic depolarization-dependent prolonged afterhyperpolarization and synaptic depression, to reduced activity resulting from a fulfilled expectation of repetition (for review, see^12^).

*In-vitro* slice studies showed that fatigue-related mechanisms of adaptation are affected by neuromodulators. Particularly, acetylcholine decreases the prolonged afterhyperpolarization that depends on potassium channels and reduces synaptic depression (reviewed in)^13^. When the fatigue mechanisms revealed in the above *in-vitro* studies contribute to repetition suppression, one would expect that repetition suppression decreases when acetylcholine is augmented. In line with this hypothesis, local intracerebral application of acetylcholine has been reported to reduce but not abolish adaptation in anesthetized rodent colliculus inferior^14^ and somatosensory (barrel) cortex^15^. In contrast to these studies in non-visual regions of rodents, a human fMRI study^16^ showed increased repetition suppression in inferior occipital cortex for faces with systemic application of the cholinesterase inhibitor physostigmine, and no region showed an attenuation of repetition suppression under physostigmine. Miller and Desimone^17^ observed no effect of systemic application of the muscarinic receptor antagonist scopolamine on repetition suppression of single macaque IT neurons. This contrasts with the increased adaptation during iontophoretic application of scopolamine observed in the rodent colliculus inferior^14^. In summary, previous studies showed cholinergic modulations of repetition suppression, but the sign of the effects differed between studies. Because acetylcholine modulates fatigue-related adaptation mechanisms^13^, we examined whether local intracortical injection of acetylcholine would affect repetition suppression in macaque IT and assessed whether it would decrease repetition suppression.

In addition to modulation by acetylcholine, rodent and human studies showed that repetition suppression can be modulated by GABA. Blocking GABA_A_ receptors reduced but not abolished repetition suppression in the anesthetized rat inferior colliculus^18^ and the medial geniculate body^19^. A role of GABAergic inhibition in repetition suppression is also in line with the decreased adaptation in auditory cortex when optogenetically suppressing inhibitory interneurons^20^. Contrary to these findings in the auditory pathway, long duration visual contrast adaptation in primary visual cortex is not affected by blocking GABA_A_ receptors^21–23^. Human fMRI studies that employed systemic application of lorazepam, which increases GABAergic inhibition, showed an increased long term repetition suppression in a task in which multiple intervening visual stimuli were present between the first and second repetitions of a stimulus, but this depended on the stimulus category and task, being absent for face repetitions^24^. Given the critical role of GABAergic inhibition in local network processing and evidence that repetition suppression in IT is a network property^25^, we determined whether blocking GABA_A_ receptors affects repetition suppression in IT.

**Figure 1:**
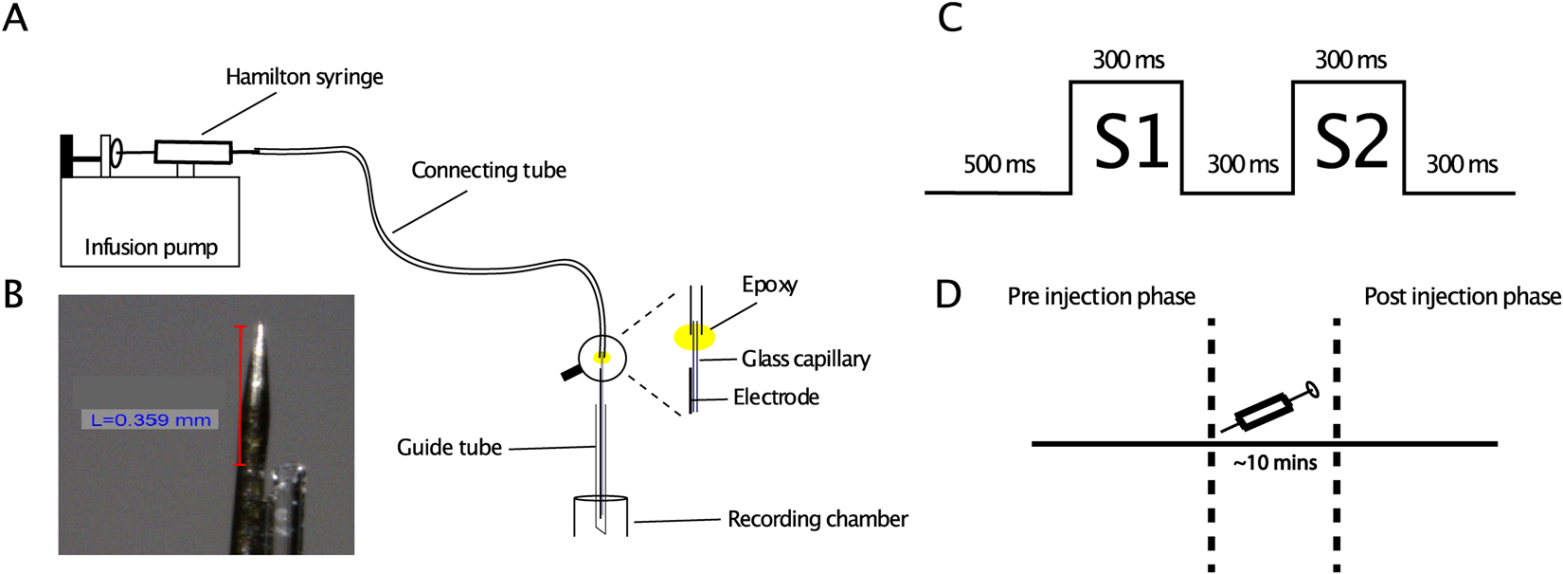
Injection set-up and experimental design. (**A**) Set-up for pressure injection. A glass capillary was glued to a metal microelectrode and was attached to a plastic tube that was connected with a Hamilton syringe which was driven by an infusion pump. (**B**) Magnified view of the tip of the tungsten microelectrode and capillary. In this example, the distance between the two tips was 359 µm, which was typical for our custom-made injectrodes. (**C**) In each trial, two stimuli were presented each for 300 ms with an ISI of 300 ms during passive fixation. The first stimulus (S1) could be the same or different as the second one (S2). (**D**) Multi-unit activity and LFPs were measured before (pre-injection phase) and after (post-injection phase) the drug injection (indicated by a syringe).

We measured multi-unit spiking activity and local field potentials before and after pressure injection of acetylcholine (ACh), the GABA_A_ antagonist Gabazine (GAB) and saline as control. The neural responses were tested using an adaptation paradigm in which two stimuli, S1 and S2, were shown successively, separated in time by a 300 ms interval (Fig. 1). The two stimuli of a sequence were either identical (repetition trials) or different (alternation trials). We compared the neural responses to S1 and S2 in the two types of trials before and after drug injections.

## Results

We assessed the effect of local intracortical pressure injection of ACh and the GABA_A_ receptor antagonist GAB on repetition suppression of spiking activity and local field potentials in IT of two monkeys. Table 1 shows the concentrations and injected volumes. First, we will describe the effects of both drugs on spiking activity, followed by a description of the drug effects on LFP power.

**Table 1:** Specifications of drug delivery.

	Concentration	Volume (uL)	Rate (uL/min)
ACh	0.05 M, 0.1 M, 0.2 M, 2 M	0.2, 0.3, 0.5	0.05, 0.1
GAB	0.04 mM, 0.06 mM, 0.3 mM, 3 mM	0.2, 0.5	0.05, 0.1
Saline	—	0.2, 0.5	0.05, 0.1

### Spiking activity: effect of ACh

**Figure 2:**
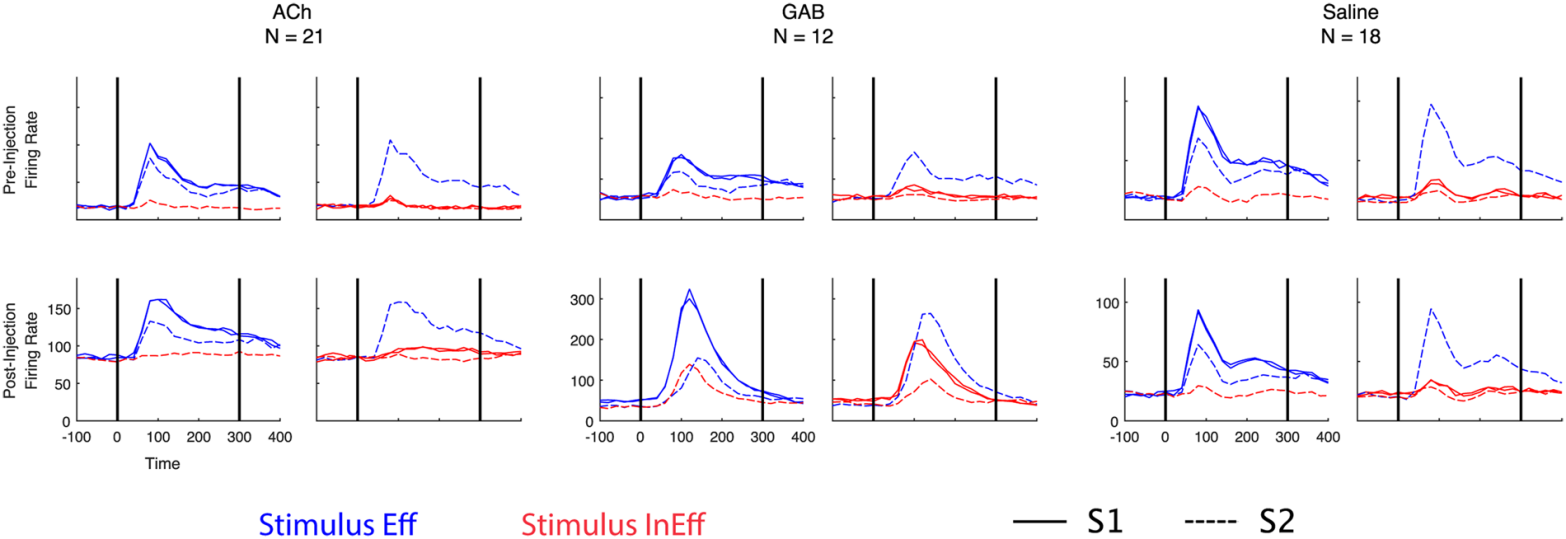
Population spiking activity before and after drug injections. The mean firing rate to S1 and S2 are indicated by full and stippled lines. For each multi-unit site, we selected two stimuli, an effective (Eff,blue) and a lesser effective (InEff, red). Each panel shows the responses in two conditions. Left panels: Eff following Eff and InEff following Eff; right panels: Eff following InEff and InEff following InEff. Since in each panel, S1 was the same stimulus in the two conditions (Eff in the left panel and InEff in the right panel) and produced a highly similar response, they are indicated with the same line format. The top panels show the average firing rate before drug injection and the lower panels the average firing rate after injection. The responses are plotted relative to stimulus onset (0) and the stimulus duration is indicated by the two vertical lines. Data were pooled across animals. Bin width 20 ms and no smoothing was applied. Left panels: effect of Acetylcholine (ACh) injections; middle panels: effect of Gabazine (GAB) injections; right panels: effect of saline (control) injections. Supplementary Figure 1 shows the same pre-injection data with an equated ordinate scale across the three drug conditions.

Figure 2 shows population PSTHs before and after injection of ACh for 4 stimulus sequences: repetition of an effective stimulus (Eff-Eff), repetition of an ineffective stimulus (InEff-InEff) and the two possible alternations of these two stimuli, Eff-InEff and InEff-Eff. The two stimuli Eff and InEff differed between the multi-unit sites and were selected for each site based on a separate Search test that was run before the injections (see Methods). Before injection of ACh, repetition of the effective stimulus produced repetition suppression: a marked reduction of the mean response to Eff following Eff. As is typical in IT^5^, the mean response to Eff when it followed the ineffective stimulus InEff was similar to Eff presented as S1. Note the highly similar responses to Eff when presented as S1 in the Eff-Eff and Eff-InEff sequences, which indicates the high reliability of the mean responses. Application of ACh increased the overall activity of the neurons. The stimulus selectivity was preserved during ACh applications, with the neurons still responding more strongly to Eff than to InEff. Overall, the responses to Eff and InEff and their time courses were highly similar before and after injection.

**Figure 3:**
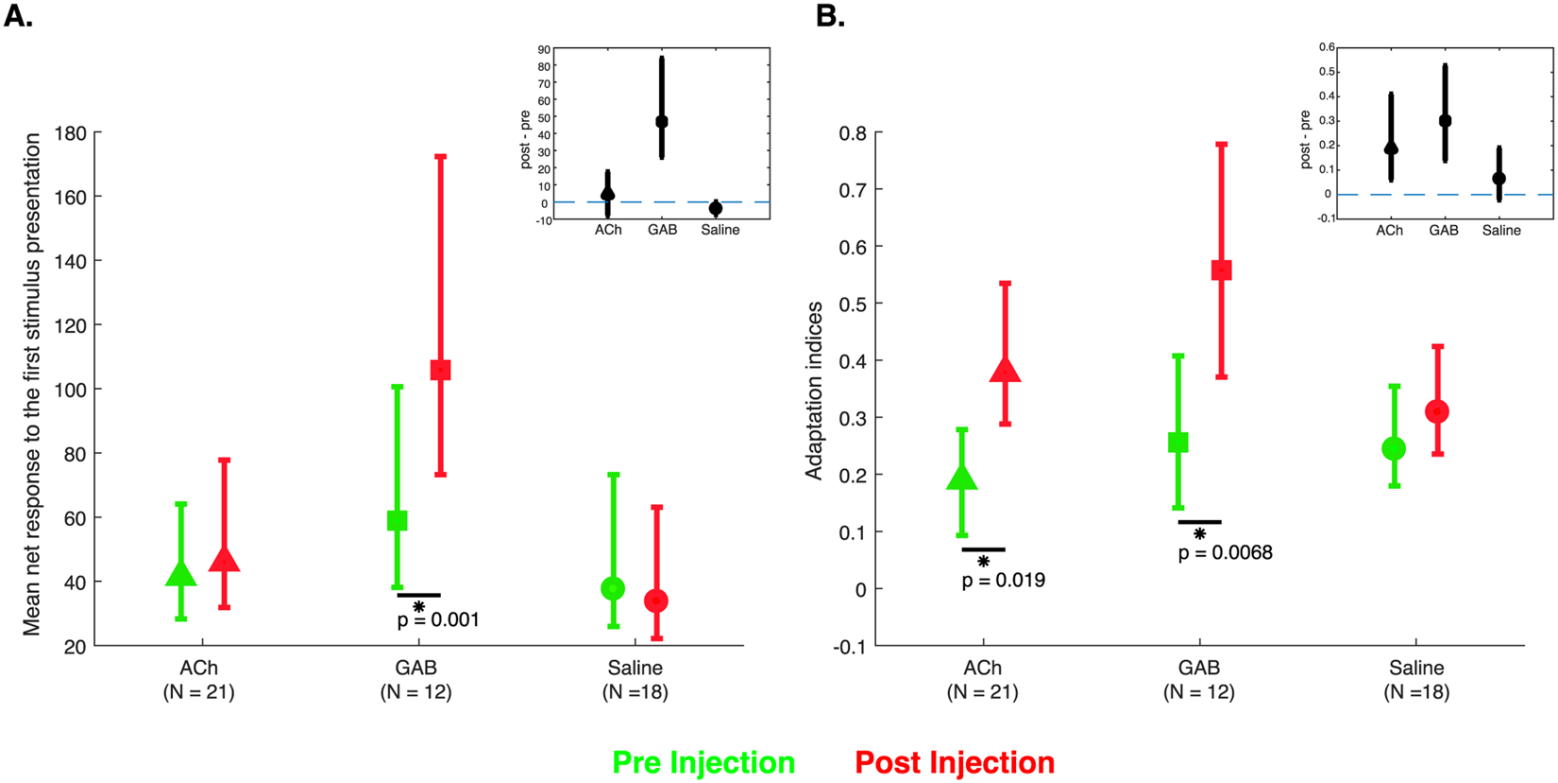
Quantification of effect of the drugs on mean firing rate and repetition suppression. (**A**) Effect of drug on mean net firing rate to S1 when stimulus Eff was presented. Error bars correspond to 95% confidence intervals computed using bootstrapping. Green and red symbols indicate the mean net firing rate measured before and after injection, averaged across MUA sites. Comparisons with a statistical significant difference between pre- and post-injection phases (Wilcoxon signed rank test) are indicated by stars and corresponding p values. The inset shows differences between mean firing rates of the pre- and post-injection phases, computed per site and then averaged across sites. Error bars show bootstrapped 95% confidence intervals of the mean difference. ACh: Acetylcholine; GAB: Gabazine; Saline: control injections with saline. (**B**) Effect of drug on repetition suppression when repeating stimulus Eff, quantified with the adaptation contrast index. Positive values correspond to repetition suppression while zero corresponds to an equal response to Eff presented as S1 and S2. Inset shows differences between adaptation contrast indices of the pre- and post-injection phases, computed per site and then averaged across sites. Errors bars are 95% confidence intervals of the mean. Same conventions as in A.

We quantified the responses to Eff, presented as S1 and S2, by computing baseline-subtracted, net responses (see Methods). The stimulus-driven activity was computed using a window of 300 ms, delayed by 60 ms after stimulus onset. Figure 3A shows the mean net responses and bootstrapped confidence intervals to Eff presented as S1 before and after ACh injections. The mean net responses to S1 were statistically indistinguishable before and after ACh injections (Wilcoxon signed rank test: p = 0.52; n = 21), indicating similar stimulus-driven responses despite the overall increase in (baseline) activity. However, the mean adaptation contrast index (see Methods; Fig. 3B) was significantly higher after compared to before ACh injection (Wilcoxon signed rank test: p = 0.019; n = 21), indicating an increased repetition suppression when applying ACh. The effect of ACh on repetition suppression can also be seen when examining the bootstrapped, within-site, post-pre injection differences in adaptation indices, which were above zero (inset in Fig. 3B). These effects on repetition suppression were consistent in the two animals (Table 2). We observed no significant correlation between the ACh-induced change in baseline activity (Post injection baseline firing rate – pre injection baseline firing rate)/(pre injection baseline firing rate) and the difference in pre- and post-injection adaptation contrast index (Spearman Rank correlation = −0.17; p = 0.46; n = 21). Thus, the change in repetition suppression with ACh appears to be unrelated to the change in baseline activity and likely reflects different mechanisms.

**Table 2:** Mean Adaptation Contrast Indices of spiking activity and confidence intervals (CI) for each monkey for pre- and post injections.

			Pre	Post
Monkey K	ACh	Mean	0.205	0.436
		CI	0.004–0.330	0.262–0.706
	GAB	Mean	0.309	0.692
		CI	0.143–0.483	0.465–0.860
	Saline	Mean	0.230	0.294
		CI	0.157–0.377	0.199–0.459
Monkey G	ACh	Mean	0.178	0.335
		CI	0.069–0.308	0.249–0.524
	GAB	Mean	0.150	0.259
		CI	0.099–0.202	0.168–0.350
	Saline	Mean	0.280	0.347
		CI	0.187–0.368	0.259–0.445

### Spiking activity: effect of Gabazine

The effect of GAB on the spiking activity of IT neurons differed markedly from that of ACh. As shown in Figs 2 and 3, GAB increased strongly the stimulus-driven firing rate (Wilcoxon signed rank test; p = 0.001; n = 12). This was true for both the effective and ineffective stimuli. Despite these strong increases in net response to both the Eff and InEff stimuli, the neurons fired more to Eff then to InEff with GAB, preserving the stimulus preference of the MUA site. The stimulus-driven responses during GAB became more transient, dropping to baseline just after stimulus offset, which is unlike the prolonged sustained responses that outlast the stimulus before the injections and which is typical in IT (see pre-injection PSTHs in Fig. 2).

The spiking activity in IT showed stronger repetition suppression with GAB application (Wilcoxon signed rank test; p = 0.007; n = 12; Fig. 3B). Notably, this greater adaptation was highly stimulus specific since presentation of Eff after InEff showed less suppression than the repetition of Eff, despite the increased and strong response to InEff (Fig. 2). Thus, the strong suppression to S2 in repetition trials with GAB is not a mere consequence of S2 being presented shortly after S1. The response onset for S2 was also delayed relative to S1. The stronger repetition suppression with GAB was evident in each animal (Table 2).

### Spiking activity: control saline injections

Although the effects on the responses differed between ACh and GAB (Fig. 3), we wanted to rule out conclusively the possibility that the response modulations were merely related to the pressure injection of a liquid volume. Thus, we injected the same volumes of saline and employed the identical pre-post injection protocol as for GAB and ACh. As shown in Figs 2 and 3, the saline injections failed to result in an effect on response strength and repetition suppression was statistically indistinguishable before and after the saline injection (Wilcoxon signed rank test; p = 0.08; n = 18). The quantitative data for the control saline injections are presented for each individual animal in Table 2.

### LFP power: effect of ACh

**Figure 4:**
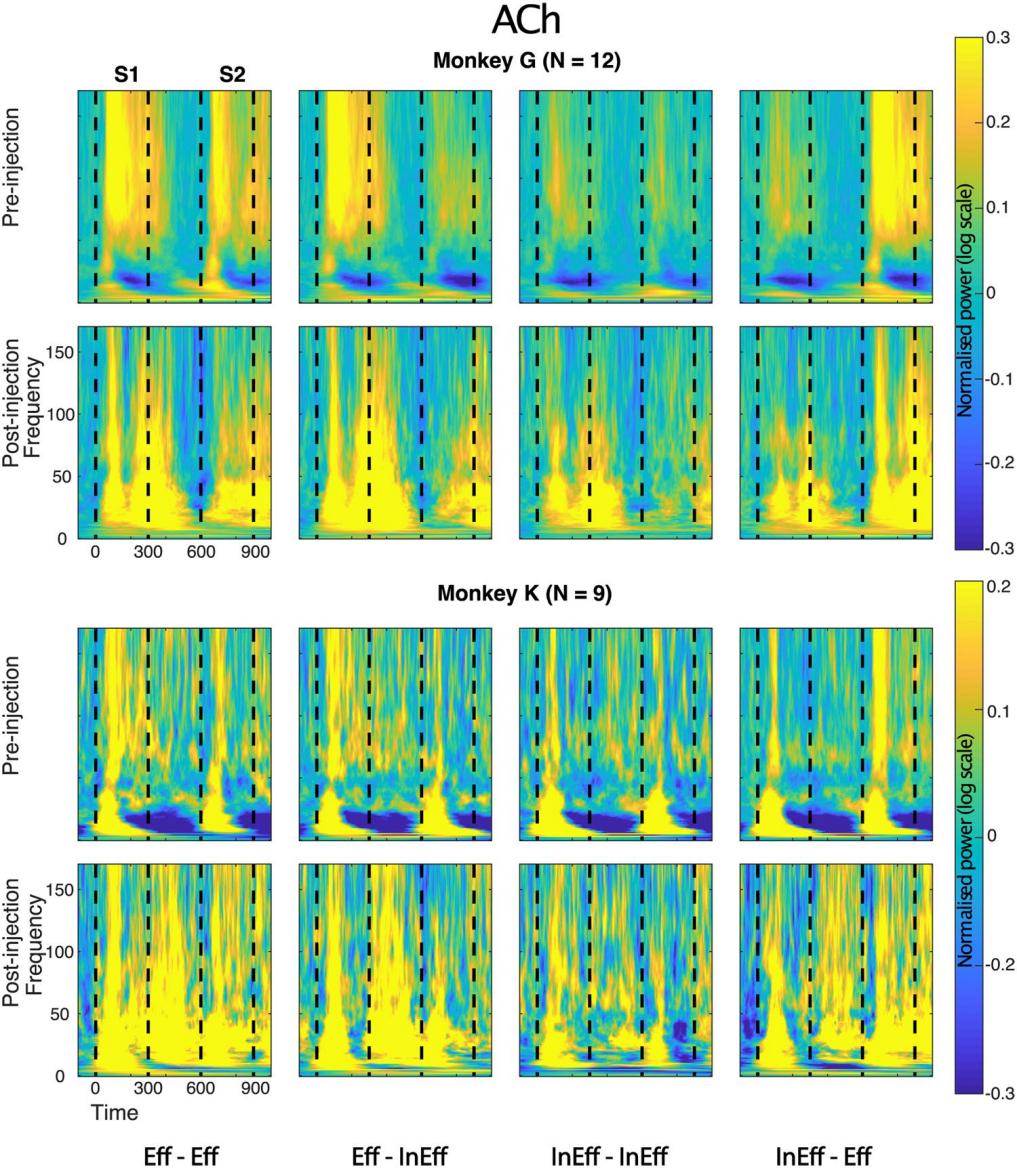
Time-frequency LFP power plots before and after Acetylcholine injection. LFP power was normalized by division by baseline power and then for visualization purposes log (base 10) transformed (a log value of 0 corresponding to a power equal to baseline). Mean normalized power was computed per site and then averaged across sites. Time-frequency plots are presented for the 4 conditions column wise (from left to right: Effective (Eff) following Effective (Eff-Eff); ineffective (InEff) following effective (Eff-InEff); ineffective following ineffective (InEff-InEff) and effective following ineffective (InEff-Eff)). The presentation of S1 and S2 are indicated in each panel by dashed vertical lines, with 0 corresponding to onset of S1. Data for the two monkeys are shown separately.

**Figure 5:**
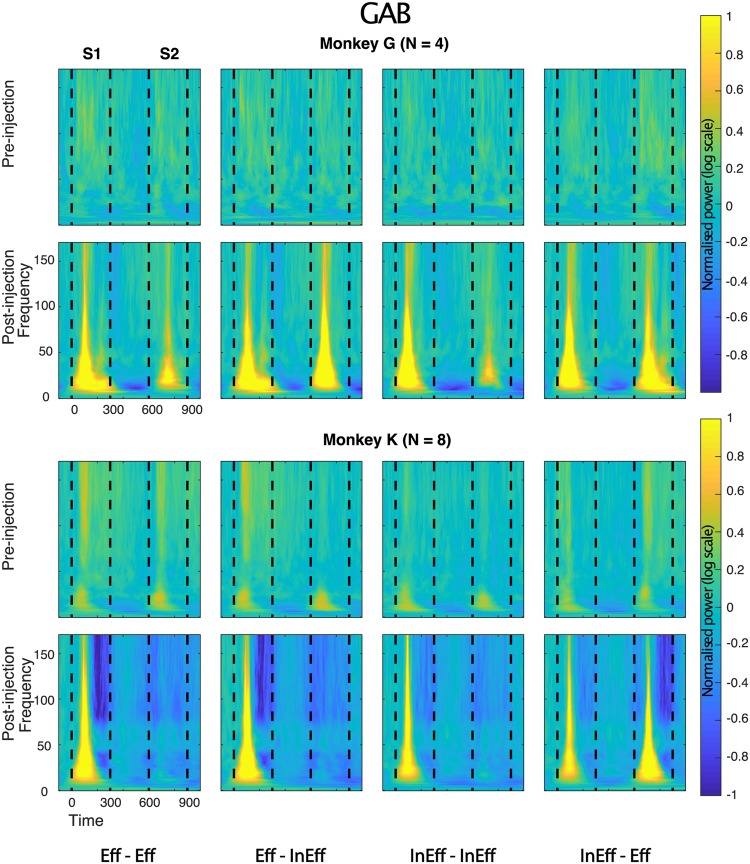
Time-frequency LFP power plots before and after Gabazine injection. Same conventions as in Fig. 4. Note that because of the strongly increased power post-Gabazine injection, in particular for the frequencies below 50 Hz, the power scale needed to be expanded compared to Figs 4 and 6. Since the same power scale was employed for the plots before and after the injection, the pre-injection power changes are less clear. However, it illustrates clearly the marked increase in power before versus after injection. Supplementary Figures S2 and S3 show the same pre-injection data with equated color scale across the three drug conditions.

**Figure 6:**
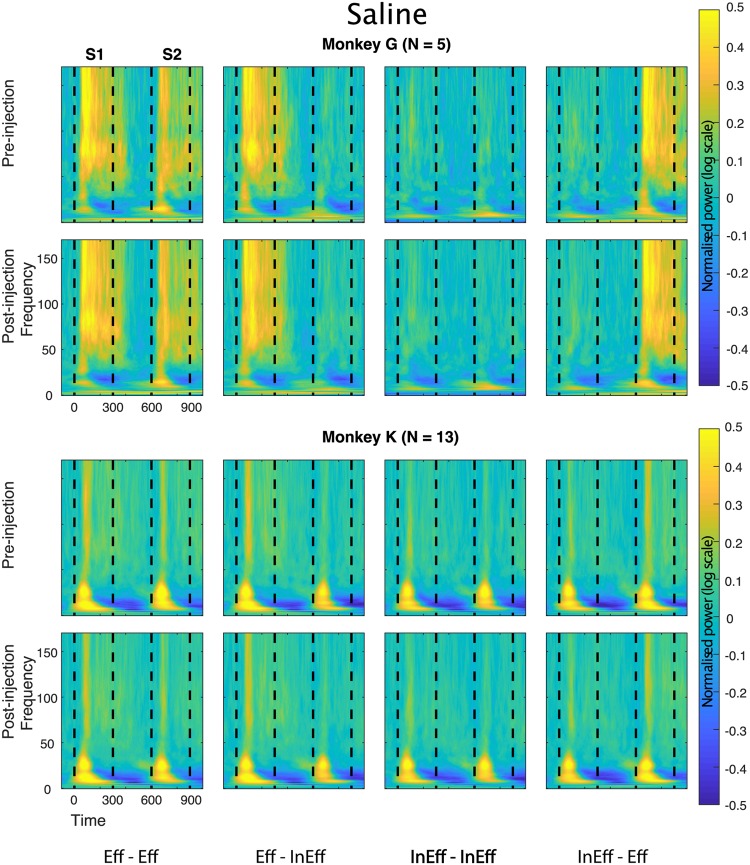
Time-frequency LFP power plots before and after saline injection. Same conventions as in Fig. 4.

**Figure 7:**
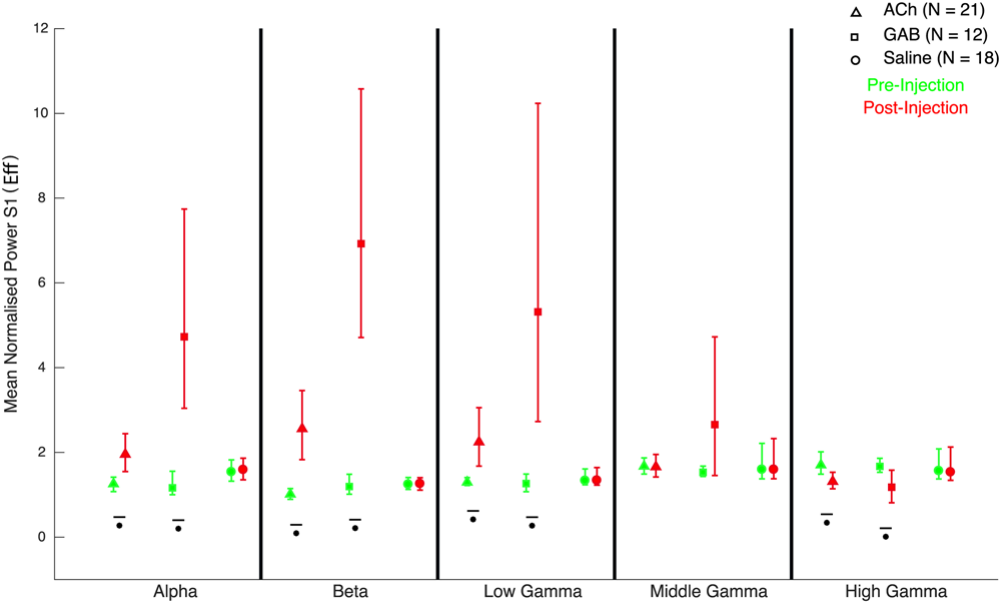
Quantification of effect of the drugs on mean LFP power. Effect of drug on mean normalized LFP power to S1 when stimulus A was presented for 5 frequency bands. Error bars correspond to 95% confidence intervals computed using bootstrapping. Green and red symbols indicate the mean normalized power measured before and after injection, averaged across sites and monkeys. Power was normalized by division by baseline but not log transformed so that a value of 1 corresponds to a power equal to baseline. Comparisons with a statistical significant difference between pre- and post-injection phases (Wilcoxon signed rank test) are indicated by stars (p < 0.05). ACh: Acetylcholine; GAB: Gabazine; Saline: control injections with saline.

**Figure 8:**
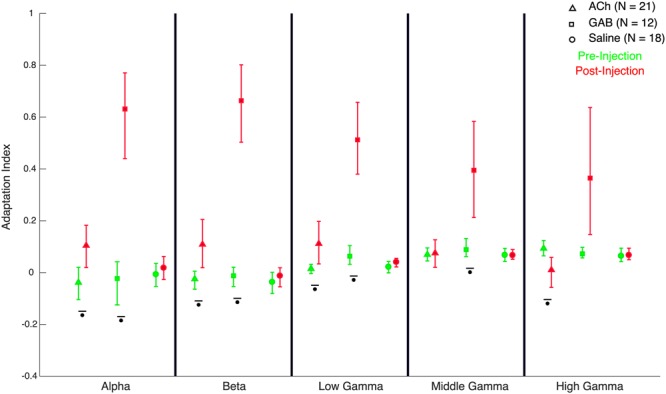
Quantification of effect of the drugs on repetition suppression of LFP power in different frequency bands. Effect of drug on repetition suppression of LFP power when repeating stimulus Eff, quantified with the adaptation contrast index. Positive values correspond to repetition suppression, negative to repetition enhancement and a value of zero corresponds to an equal response to Eff presented as S1 and S2. Error bars correspond to 95% confidence intervals computed using bootstrapping. Comparisons with a statistical significant difference between pre- and post-injection phases (Wilcoxon signed rank test) are indicated by stars (p < 0.05). Same conventions as in Fig. 7.

As in our previous studies of repetition suppression in IT^10,11^, repetition suppression was present before injection in the LFP power for frequencies above 50 Hz, while absent for alpha and beta power (Fig. 4; data shown for individual monkeys). Note that the LFP power shown in Figs 4–6 was normalized relative to baseline and thus reflects stimulus-induced changes in power and not absolute power. Thus, possible drug-induced absolute changes in power, unrelated to the stimuli, are removed. Application of ACh mainly affected frequencies below 50 Hz, producing an increased power in the alpha, beta and low gamma ranges (Figs 4 and 7). In both monkeys (Fig. 4) and typical for IT, the pre-injection power in alpha and beta ranges decreased below baseline values after an initial transient activation. In both animals, ACh abolished this de-activation and instead produced a marked power increase in that time period that lasted until 200 ms into the ISI. This lead to an increased power (averaged across the 300 ms analysis window) post- compared to pre-injection of ACh in the alpha, beta and low gamma bands (Fig. 7). Furthermore, application of ACh resulted in repetition suppression in these low frequency bands, which was absent before the injection (Fig. 8; for statistics per power band, see Fig. 8). Repetition suppression in the mid-gamma band was not significantly affected by ACh application but there was a significant reduction of repetition suppression for the high gamma band when the power was averaged across the stimulus presentation window (Fig. 8). Inspection of the time-frequency plots (Fig. 4) showed that this reduction of repetition suppression in the high gamma bands was largely due to the more sustained time course of the power for the S2 stimulus in the Eff-Eff sequence. Note that in both animals (Fig. 4), the initial response to the S2 stimulus was still suppressed compared with the response to the same stimulus presented as S1 in the Eff-Eff sequence. Quantitative data for each monkey are presented in Table 3.

**Table 3:** Mean Adaptation Contrast Indices and confidence intervals (CI) computed for LFP power per frequency band for each monkey, pre- and post injections.

			ACh		GAB		Saline	
			Mean	CI	Mean	CI	Mean	CI
Monkey K	Alpha	Pre	0.062	−0.004–0.126	−0.057	−0.179–0.049	0.039	−0.003–0.071
		Post	0.092	−0.065–0.192	0.655	0.455–0.813	0.062	0.021–0.101
	Beta	Pre	−0.008	−0.04–0.041	−0.034	−0.085–0.016	−0.024	−0.061–0.017
		Post	0.094	−0.041–0.238	0.756	0.568–0.893	0.003	−0.02–0.04
	Low Gamma	Pre	0.034	0.012–0.054	0.096	0.060–0.142	0.034	0.008–0.057
		Post	0.115	−0.008–0.215	0.594	0.427–0.791	0.05	0.039–0.064
	Middle Gamma	Pre	0.079	0.042–0.119	0.106	0.067–0.163	0.059	0.036–0.086
		Post	0.078	−0.003–0.119	0.538	0.331–0.764	0.057	0.042–0.073
	High Gamma	Pre	0.068	0.024–0.110	0.08	0.058–0.118	0.048	0.031–0.068
		Post	−0.039	−0.139–0.032	0.547	0.271–0.839	0.053	0.036–0.075
Monkey G	Alpha	Pre	−0.113	−0.18–−0.023	0.045	−0.007–0.071	−0.128	−0.186–−0.074
		Post	0.114	0.029–0.225	0.584	0.216–0.795	−0.097	−0.148–−0.066
	Beta	Pre	−0.037	−0.093–0.008	0.031	0.007–0.055	−0.068	−0.167–0.011
		Post	0.12	0.019–0.279	0.478	0.238–0.619	−0.054	−0.157–0.022
	Low Gamma	Pre	0.001	−0.022–0.027	−0.002	−0.005–−0.0002	−0.009	−0.04–0.033
		Post	0.109	0.008–0.255	0.348	0.206–0.584	0.015	−0.028–0.051
	Middle Gamma	Pre	0.063	0.031–0.097	0.055	0.049–0.060	0.091	0.039–0.144
		Post	0.073	−0.005–0.161	0.108	−0.071–0.389	0.093	0.058–0.144
	High Gamma	Pre	0.112	0.085–0.151	0.056	0.054–0.059	0.107	0.042–0.167
		Post	0.047	−0.069–0.1	0.001	−0.205–0.076	0.105	0.066–0.162

### LFP power: effects of Gabazine

In both animals, injection of GAB produced a marked increase in stimulus-driven power, particularly for the frequencies below 100 Hz. This power increase was pronounced and statistically significant in the alpha, beta and low gamma bands for S1 (Figs 5 and 7). Notably, the power increase in these low frequency bands with application of GAB was greater for S1 compared with S2 in repetition trials (Fig. 7), which resulted in a highly and significantly increased repetition suppression in these bands (Figs 5 and 8). Repetition suppression also increased significantly for the mid gamma band but the increase failed to reach statistical significance for the high gamma bands (Fig. 8). The increased repetition suppression with GAB application was not merely a result of suppressed responses to any stimulus that followed another one closely in time since the response to Eff following InEff in the InEff-Eff sequences was similar to that to Eff presented as S1 in Eff-Eff or Eff-InEff sequences (Fig. 5). In both animals, the power in the gamma bands dropped below baseline after the initial response transient after injecting GAB (Fig. 5). This late inactivation was present for the S1 stimuli and when Eff was presented as an S2 stimulus in alternation trials (InEff-Eff). Note that such a below baseline activity was not seen in the simultaneously recorded spiking activity (Fig. 2), implying that the gamma response measured in this study does not merely reflect spike intrusion (see^10,26^ for another discrepancy between high-frequency gamma power and concurrently measured spiking activity related to repetition suppression and^10^ for a discussion of the role of spike intrusion to gamma band power measured with the same methods as here).

### LFP power: control saline injections

Control experiments with saline injection did not produce significant effects on the S1 power (Figs 6 and 7) and repetition suppression was statistically indistinguishable before versus after injection for all 5 spectral bands (Fig. 8; Table 3).

## Discussion

Both application of Ach and the GABA_A_ antagonist Gabazine increased repetition suppression of macaque IT spiking activity. In both cases, repetition suppression was stimulus selective, with little suppression for an effective stimulus when it was preceded by a less effective stimulus. This was even true in the case of Gabazine, which increased greatly the response to a stimulus that produced only a small response before the drug application. Increased repetition suppression was present also for LFP power after injections of ACh and GAB, particularly at frequencies below 50 Hz for which typically no repetition suppression is present in macaque IT.

The effects of both drugs were consistent in the two animals and across individual sessions. We presented here data for those sessions in which we had both pre- and post-injection measurements. Particularly for GAB, we have also recordings for which no pre-injection data were collected. These data were obtained from neighboring sites in the same daily sessions after the pre- and post-injection data of an injection site were collected. This was possible since the effect of GAB was present during an entire recording session, at these sites (n = 44) also, marked repetition suppression was present when applying GAB (mean adaptation contrast index = 0.45, significantly greater than 0 (Wilcoxon test: p < 0.0001)). The repetition suppression at these 44 sites with GAB was significantly greater than the one obtained pre-injection at neighboring but not identical sites (mean adaptation contrast index = 0.22; n = 51 (pre-injection sites for GAB, ACh and saline pooled); Mann-Whitney U test: p = 0.001).

Repetition suppression was absent (ACh) or weak (GAB) when adapter and test stimuli differed. This implies that the stronger suppression for the test stimuli is not a mere stimulus sequence effect but depends on the identity of adapter and test stimulus, as does repetition suppression under no-drug conditions^5,10^.

The drug effects we observed here in awake macaque IT cortex are opposite to those reported in rodents in other sensory systems. Indeed, for both ACh^14,15^ and GAB^18,19^, previous studies in rodents showed decreased stimulus selective adaptation. In these studies, repetition suppression was still present with drug application and it is unclear whether the smaller adaptation indices did not result from increased responses per se since raw instead of net responses were employed to compute adaptation indices (except for one control analysis in^14^). There are multiple differences between our study in the visual system of alert macaques and the studies in anesthetized rodents in other sensory modalities. Hence, it is difficult to know whether the discrepancy is due to a difference between species, brain regions, brain state, technique (pressure injection versus iontophoresis), adaptation paradigm (e.g. oddball versus single trial adaptation) or affected brain volume.

Despite the major differences in drug application methodology, adaptation paradigms and neural measurements, human pharmacological neuroimaging studies suggested modulations of repetition suppression with actelylcholine^24^ that are in line with the present findings in monkey IT. The effects reported in human fMRI and the present in macaques are opposite to what one predicts based on the *in vitro* effects of ACh on prolonged afterhyperpolarization and synaptic depression^13^. One possible explanation is that both these fatigue mechanisms have locally in IT a minor contribution to repetition suppression. Indeed, it is possible that repetition suppression in IT mainly results from suppressed inputs of other IT neurons not affected by the drug and neurons upstream to the ones we recorded from. Thus, the increased repetition suppression with ACh application in the neuronal sites we recorded from may have resulted from increased activation of these neurons by adapted upstream input. Indeed, we observed a marked increase in LFP power in the low frequency bands (below 50 Hz), which are thought to reflect synaptic input and not spiking activity^27^. This increased activation by the upstream input may have resulted from an ACh-induced decreased inhibition through a reduced efficacy of GABAergic synapses (reviewed in^28^). Unlike without drug application, the low frequency bands showed repetition suppression with ACh, in agreement with a transsynaptic origin of the repetition suppression.

GABA-related effects on repetition suppression as assessed with fMRI in humans are highly variable between tasks^24^ and thus difficult to compare with our monkey data. Applying a GABA_A_ antagonist increased spiking responses to the stimuli, reduced the stimulus selectivity and increased repetition suppression of spiking activity in macaque IT. In addition, there was a profound increase of the LFP power for the low frequencies which showed strong, stimulus-specific repetition suppression. Because LFP power in these low frequencies mainly reflect synaptic input, the most parsimonious explanation of our data is that the repetition suppression resulted from input from upstream areas and from other neurons in IT. The repetition suppression at the low frequencies then reflects the suppressed input from the upstream and other IT neurons following repetition. The decreased inhibitory input produced both enhanced excitatory input from neighboring neurons as well increased the impact of upstream excitatory input which is normally at least partially inhibited. We know from previous studies that IT neurons receive input from other neurons that is normally masked by inhibitory input^29,30^. Preservation of the stimulus selectivity of repetition suppression with GAB application can result from input, from neighboring sites and/or upstream areas, that is specific for the feature differences between the two stimuli. In such a scheme, the recorded neurons receive excitatory input from neurons that encode features of Eff and neurons that encode features of InEff, but the latter input is inhibited. Application of GAB reduces the latter inhibition, producing a response to stimulus InEff in the neurons. However, because the input is still largely stimulus specific, i.e. differs for stimulus features of Eff and InEff, the repetition suppression will remain stimulus specific^5^. The increased repetition suppression of the spiking activity with GAB application might result from a nonlinear relationship between spiking activity and the enhanced synaptic input that shows repetition suppression, i.e. an expansive nonlinearity between membrane potential and spikes^31^. Another, non-exclusive, possibility is that the strongly increased pre- and post-synaptic responses with GAB application causes enhanced synaptic depression or response fatigue. The latter can explain the below baseline power in the late response phase for the high frequency power as well as the more transient responses (reflecting adaptation during the response) that we observed with GAB application.

The absence of a reduced repetition suppression in IT with application of a GABA_A_ antagonist is in line with studies that showed that long duration adaptation in V1 does not depend on GABA_A_ receptors^21–23^. The ionotropic GABA_A_ receptors gate chloride channels that cause fast inhibitory postsynaptic potentials. A potential involvement in adaptation processes of GABA_A_ receptors could have resulted from inhibition from interneurons that enhance their response with repetition, stronger synaptic depression of excitatory compared with inhibitory synapses, or a comparison between stimulus predictions and stimulus input that relies on inhibitory mechanisms (similar to reward prediction errors in the Ventral Tegmentum Area^32^). The present data as well as previous work in primary visual cortex argue against such role of GABAergic mechanisms in visual adaptation and repetition suppression. GAB does not block the slower GABA_B_ receptors which as metabotropic receptors produce relatively long lasting inhibition. Adaptation to visual stimuli in the rat superior colliculus is reduced by application of a GABA_B_ receptor antagonist^33^, but contrast adaptation in primary visual cortex is not affected by GABA_B_ receptor blockage^23^. Further research is required to determine whether GABA_B_ receptors are involved in repetition suppression in macaque IT but we deem this unlikely given the absence of a role of these receptors in adaptation of primary visual cortical neurons.

In sum, repetition suppression in macaque IT cortex was enhanced when applying acetylcholine and a GABA_A_ receptor antagonist. Both drugs also enhanced LFP power and repetition suppression in low frequency bands. These findings agree with repetition suppression of an IT neuron being mainly the result of suppressed input from upstream and other IT neurons, but further work is needed to test this suggestion directly.

## Methods

### Subjects

Two rhesus macaques (*Macaca mulatta*; male monkey G and female monkey K) served as subjects. Animal care and experiments were carried out in accordance with the national and European guidelines and were approved by the Animal Ethics Committee of the KU Leuven.

Details about implants and surgery can be found in^34^ and will only be briefly summarized here. The placement of the plastic recording chamber was guided with a preoperative magnetic resonance imaging (MRI) scan and verified with MRI scans obtained postoperatively before and in-between recording sessions. For the latter MRI scans, the recording chamber, with the Crist grid located at the same position as during the recordings, was filled with a 1% solution in saline of the gadolinium-based contrast agent Gadoteric acid (Dotarem). This combined with the insertion of tungsten wires, enclosed in glass capillaries, in the grid at 5 locations enabled visualization of the recording chamber, grid and electrode trajectories. Recording positions were estimated based on the MRI visualization of these markers combined with the microdrive depth readings of the white/gray matter transitions relative to the grid base.

### Recordings and injections

Simultaneous recordings and pressure injections were performed with custom-made injectrodes (Fig. 1). We glued a thin quartz capillary (CM Scientific; inner diameter (ID) = 50 um; outer diameter (OD) = 80 um) to a 125 µm diameter tungsten microelectrode (FHC; impedance of ~1MOhms). One end of the glass capillary was glued close to the tip of the electrode (<0.5 mm distance between electrode tip and capillary) while the other end was attached airtight to a polyethylene tube (Intramedic; ID = 380 um; OD = 1090 um) with dental base resine (Lucitone 199). This tube was connected to a needle (OD: 400 um) of a 5 uL Hamilton syringe. The syringe was driven by a commercial infusion pump (Chemyx Syringe Pump Fusion 710), allowing us to manipulate precisely the injection rate and volume of the drug administration (Table 1). With these volumes, we expect that the drugs affected at least a 10 mm^2^ area^35^.

The injectrode was lowered with a custom modified Narishige microdrive through a guide tube that was fixed in a Crist plastic recording grid. The guide tube was grounded and served as a reference. Amplification and filtering of the electrical signals were performed by a Plexon data acquisition system (Plexon Inc.). Recorded signals were preamplified with a headstage having an input impedance of >1GΩ. The signal was bandpass filtered between 250 to 8000 Hz for spikes. Triggered waveforms were saved at 40 kHz for later offline analysis (Offline Sorter; Plexon). The broadband signal was also recorded simultaneously with a sampling rate of 40 kHz.

Eye position was measured online with an infrared-based eye tracking system (ISCAN EC-240A, ISCAN Inc.; 120 Hz sampling rate). The analog eye movement signal was saved with a sampling frequency of 1 kHz. In all tests, we employed fixation windows that measured maximally 2° on a side. Eye positions, stimulus and behavioral events were recorded simultaneously with spiking and broadband activity and stored for later off-line analysis on a computer that was synchronized with the Plexon data acquisition system.

### Drug Administration

Drugs were diluted to the desired concentration in 0.9% saline. Table 1 shows the employed Acetylcholine (ACh; Sigma-Aldrich) and Gabazine (GAB; Sigma-Aldrich) concentrations, injection rates and volumes. Before filling the Hamilton syringe, the drug solution was filtered by passing it through a sterile syringe filter (0.2 um Cellulose Acetate membrane; VWR International). The complete injection setup was verified in each session pre- and post-recording by visual inspection of fluid discharge through the capillary with a slight movement of the syringe plunger. Post-recording, undamaged injectrodes were cleaned with an enzyme-active detergent (Tergazyme) solution for re-use.

### Stimuli

We employed a stimulus set identical to that used in some of our previous studies on adaptation in macaque IT^34^. The stimulus set consisted of 52 color images of 26 object classes (2 images per class) including human and monkey faces, human and monkey bodies, mammals, birds, fish, snakes, insects, trees, fruits, fractals and manmade objects. The size of the stimuli (maximum of horizontal and vertical dimensions of the bounding box) was approximately 5° of visual angle. The stimuli were presented on a uniform gray background on a CRT monitor (Phillips Brilliance 202P4, frame rate = 60 Hz, resolution = 1024 × 768 pixels) located approximately 60 cm from the subject’s eyes.

### Tests

#### Search Test

While advancing the injectrode in IT, we searched for responsive neurons using a search test. On each trial of the search test, the monkeys were required to maintain their gaze on a red fixation target square (size = 0.17°) presented in the center of the monitor and visible during the entire trial. A trial started with the onset of the fixation target. After 300 ms of stable fixation, a stimulus was presented for 300 ms. To complete a trial and obtain a fluid reward, the monkeys had to maintain fixation for 300 ms poststimulus. The 52 images were presented foveally in a random order. At a responsive multi-unit site, the search test was used to select two stimuli, with one of the two stimuli evoking a strong response (effective stimulus, labeled Eff) and the other little or no response (ineffective stimulus, labeled InEff). The two stimuli were selected online from the pool of 52 color images by inspection of post-stimulus time histograms (PSTH) of the responses to each of the stimuli.

#### Adaptation test and injection protocol

We examined the effect of drug administration on adaptation with the following adaptation test (Fig. 1). After the onset of the fixation target, the monkey had to fixate that target for 500 ms. After that prestimulus fixation period, a stimulus (S1) was presented for 300 ms, which served as an adapter. The fixation target was presented on top of the centrally presented stimulus. After the presentation of the adapter stimulus, there was an interstimulus interval of 300 ms during which only the fixation target was presented. The interstimulus interval was followed by the presentation of the test stimulus (S2) for 300 ms. As for the adapter stimulus, the fixation target was presented on top of the test stimulus. The test stimulus was presented at the same location as the adapter stimulus. After the offset of the test stimulus, the fixation target was presented for another 300 ms. Continuous fixation during the whole trial was rewarded with a liquid reward after the 300 ms post-test fixation period. After delivering the reward, we presented a full-field scrambled stimulus for 500 ms followed by a blank screen for 150 ms. Each scene was scrambled five times (20 scenes × 5 = 100 stimuli). During this period, the monkey was not required to fixate. The presentation of the scrambled stimulus served to reduce the carry-over of adaptation effects from the adapter and test stimuli to the subsequent trial. The median inter-trial interval (time interval between S2 offset and S1 onset of the next trial) was 2440 ms (minimum = 2206 ms; 95^th^ percentile = 5399 ms).

S1 and S2 could be either Eff or InEff with equal probability. This resulted in 4 possible types of stimulus sequences of which Eff – Eff and InEff – InEff are repetition trials and Eff- InEff and InEff – Eff are alternation trials. The 4 sequences were presented in random order.

After the search test and selecting the two stimuli, the adaptation test was run in two phases: the pre-injection and post-injection phase. In the pre-injection phase, we presented the adaptation test for a median of 49 unaborted trials per condition (minimum = 8 trials, maximum = 83 trials; N = 51 sites). Then the drug/saline was injected and after a waiting period of approximately 10 minutes, the post-injection adaptation test was presented for a median of 63 unaborted trials per condition (minimum = 8 trials, maximum = 208 trials; N = 51 sites).

Duration of post-injection sessions were different for ACh and GAB. Since the recovery of the drug effect was relatively short, approximately 30–40 mins, in the case of ACh compared to GAB, we were able to record from multiple sites (at least 500 um away from each site) during the same recording day. In the case of GAB, we did not observe a recovery of the drug effect within a recording session. Hence, we analyzed for GAB only one site per daily recording session. For saline control injections, we applied the same protocol as for ACh, i.e. post injection we recorded for approximately 30–40 mins (or until the multi-unit activity to the stimuli lasted) and then moved to a new site.

We present results for those injections for which we had both pre- and post-injection recording data for the same site. For ACh, we made in total 52 injections of which 21 were included in the analysis. In the other cases, the multi-unit activity was unstable (a decrease of the amplitude of the spikes or units being damaged by electrode) or was not responsive to the stimuli (11 cases) or the injectrode malfunctioned (capillary broke; 20 cases). For GAB we made 29 injections of which 12 provided data of the pre- and post-injection phase. Five other injections were unsuccessful because of a malfunctioning capillary and for 12 the multi-unit activity was lost or unresponsive. For the saline control injections, we made 36 injections, of which 18 could be used for further analysis, while for 8 others the capillary broke and for 10 the multi-unit activity was lost.

### Data Analysis

For each recorded multi-unit site and stimulus sequence type, we computed the mean firing rate to the adapter and test stimuli. Only unaborted trials were analyzed. Responses to the stimuli were computed within a 300 ms long analysis window starting at 60 ms after stimulus onset. In all experiments, the onset of the adapter and test stimuli were detected by a photodiode and those timings were employed to align the neural activity to S1 and S2 separately. For each multi-unit site we computed an adaptation contrast index using the net responses to A in repetition trials:

$$\frac{{\rm{R}}1-{\rm{R}}2}{|{\rm{R}}1|+|{\rm{R}}2|}$$

with R1 and R2 being the net firing rate to S1 and S2, respectively. The net firing rate was computed as the difference between the firing rate measured during stimulus presentation and the baseline activity (mean firing rate in the interval between 300 ms before the onset of S1 and the onset of S1). Peristimulus time histograms (PSTHs) were computed for each site and stimulus condition, before and after the injections, by averaging the firing rate in bins of 20 ms, aligned on stimulus onset. Population PSTHs were created by averaging the firing rate across neurons per sequence type.

Local Field Potentials (LFPs) were downsampled to 1000 Hz before performing any further analysis. LFPs were filtered offline with digital 50 Hz and 60 Hz notch filters (fourth-order Butterworth FIR filter; Fieldtrip Toolbox, F.C. Donders Centre for Cognitive Neuroimaging, Nijmegen, The Netherlands) to remove AC line noise (50 Hz) and CRT refresh rate related activity (60 Hz). Trials in which the signal was <1% or >99% of the total input range were removed as outliers, which also protected against clipping. In order to compare the adaptation effects for LFP power in different frequency bands with those of previous studies, we employed the same method for spectral analysis of the LFP as^10,11,36^. By convolving single-trial data using complex Morlet wavelets and taking the square of the convolution between the wavelet and signal, the time-varying power of the signal for every frequency was obtained. Averaging spectral maps (power as a function of frequency and time) across trials for a given stimulus condition and site produced a spectral map of that condition and site. The complex Morlet wavelets had a constant center frequency-spectral bandwidth ratio f0/σf of 7, with f0 ranging from 1 to 170 Hz in steps of 1 Hz. The spectral maps of the sites were normalized at each frequency by division by the average power computed within a baseline window of −200 ms to 0 ms before S1 onset. The window was limited to 200 ms to avoid the spectral edge effect. For a quantitative analysis of LFP power, we divided the frequency spectrum into 5 bands: alpha (8–12 Hz), beta (13–30 Hz), low gamma (31–60 Hz), middle gamma (61–100 Hz) and high gamma (100–170 Hz). As noted by^10^, power estimates using the Morlet wavelet analysis for frequencies below 15 Hz can be affected by an overlap of responses to test and adapter stimulus for the long duration (>>150 ms) wavelets at these low frequencies. This implies that changes in alpha power, in particular estimates of the absolute size of the adaptation effect, should be treated with some caution. The neuromodulatory effect of GAB lasted for several hours, i.e. a complete recording session, even at the lowest concentrations and volumes that we employed. So, to analyze the effect of GAB, we took all the data of the pre- and post-injection adaptation tests. In contrast, the recovery from ACh injection was faster and within a recording session, necessitating an additional step to select the test period where the drug affected the response. Previous studies in cortex and preliminary inspection of our data showed that ACh increases overall neural activity. Thus, the onset of the post-injection analysis period was defined as the time at which the mean neural signal strength, operationalized as the rectified broadband signal, was greater in 10 consecutive trials than the 90^th^ percentile of the signal strength in the pre-injection trials (threshold). Similarly, the end of the analysis period was defined by a trial above the same threshold that was preceded by 9 consecutive trials above the threshold. Note that this analysis period was defined without identifying the stimulus conditions (e.g. S1 versus S2) or the stimulus sequences and thus could not have biased an effect of ACh on adaptation, since the latter entails a comparison of the responses between S1 and S2. Supplementary Figure S4 illustrates the determination of the post-injection analysis period for the ACh data.

We computed the significance of the difference between stimulus conditions with a nonparametric Wilcoxon signed rank test (applied to the mean response in a window of 60 ms to 360 ms after stimulus onset). The 95% confidence intervals of mean responses, response differences and adaptation contrast indices were computed using bootstrapping (Matlab bootci function; Bias corrected and accelerated percentile method).

Data are available upon request from the authors.

## Electronic supplementary material

Supplementary Information
