# Supplementary material for: GABAergic and cholinergic modulation of repetition suppression in inferior temporal cortex
Source: Sci Rep. 2018 Sep 3;8:13160. doi: 10.1038/s41598-018-31515-1 (PMC6120963; doi:10.1038/s41598-018-31515-1)
Supplement: Supplementary file 1 — Supplementary Information [file 41598_2018_31515_MOESM1_ESM.docx]

**Supplementary Information**

**GABAergic and cholinergic modulation of repetition suppression in inferior temporal cortex.**

Pradeep Kuravi^1,2^ , Rufin Vogels^1,2^*

^1^Laboratorium voor Neuro- en Psychofysiologie, Department of Neurosciences, KU Leuven, Leuven, Belgium.

^2^ Leuven Brain Institute, Leuven, Belgium

Contents:

Figure S1.

Figure S2.

Figure S3

Figure S4.


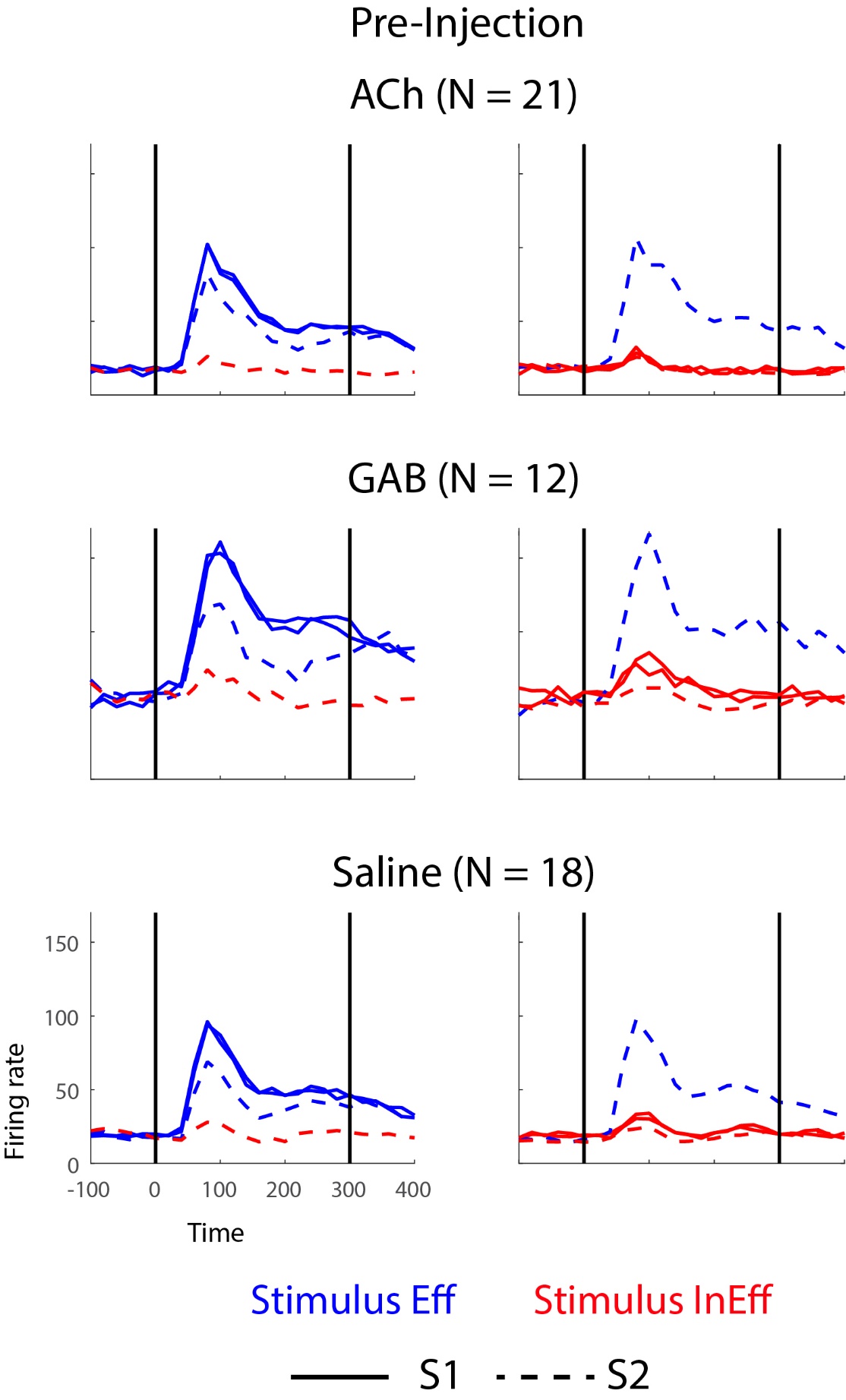


**Figure S1. Population spiking activity before drug injections**. The mean firing rate to S1 and S2 are indicated by full and stippled lines. For each multi-unit site, we selected two stimuli, an effective (Eff,blue) and a lesser effective (InEff , red). Each panel shows the responses in two conditions. Left panels: Eff following Eff and InEff following Eff; right panels: Eff following InEff and InEff following InEff. Since in each panel, S1 was the same stimulus in the two conditions (Eff in the left panel and InEff in the right panel), they are indicated with the same line format. The responses are plotted relative to stimulus onset (0) and the stimulus duration is indicated by the two vertical lines. Data were pooled across animals. Bin width 20 ms and no smoothing was applied. Top panels: effect of Acetylcholine (ACh) injections; middle panels: effect of Gabazine (GAB) injections; bottom panels: effect of saline (control) injections. The Y axis scale was equated across the 3 drug conditions.


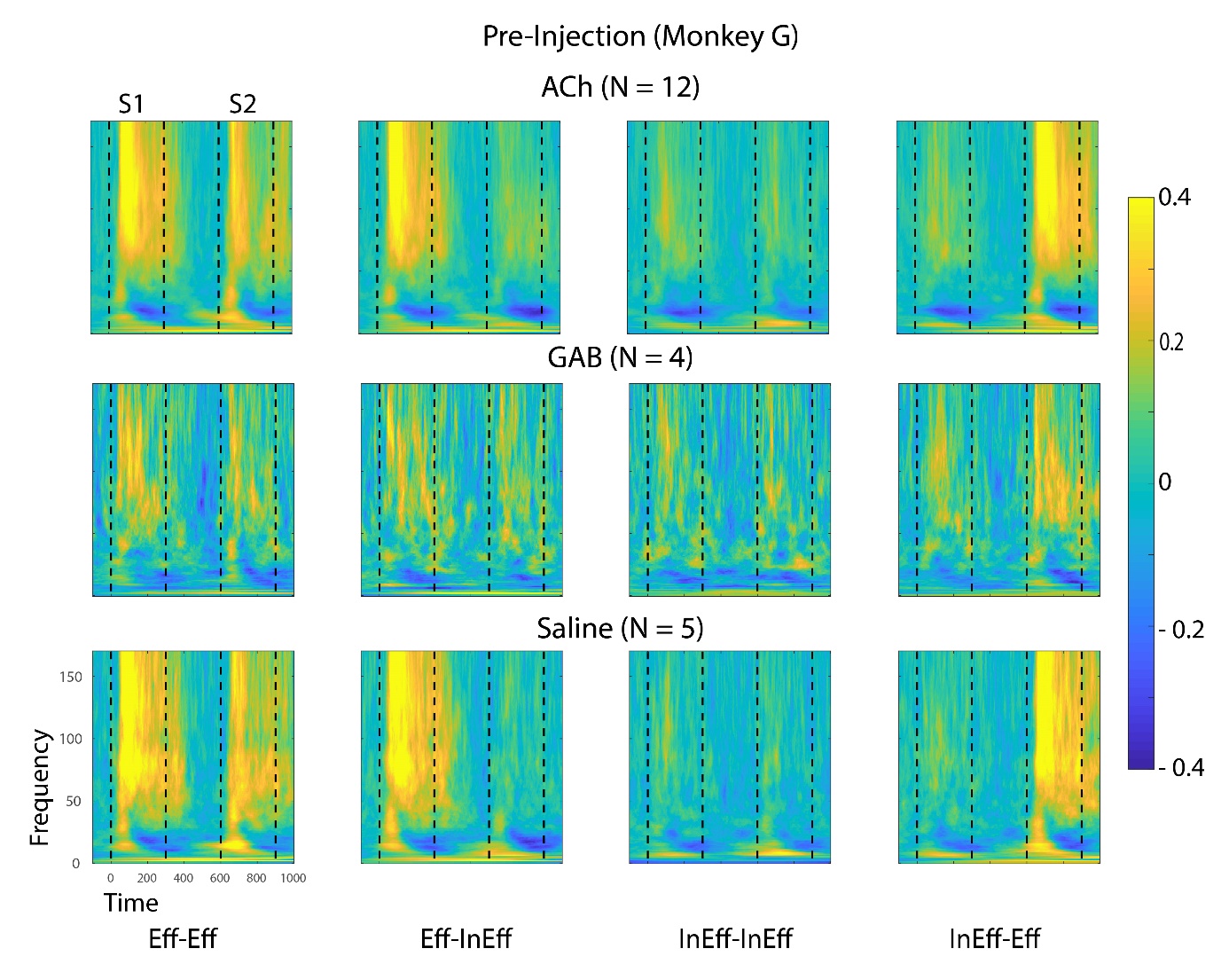


**Figure S2. Time-frequency LFP power plots before drug injections in monkey G**. LFP power was normalized by division by baseline power and then for visualization purposes log (base 10) transformed (a log value of 0 corresponding to a power equal to baseline). Mean normalized power was computed per site and then averaged across sites. Time-frequency plots are presented for the 4 conditions column wise (from left to right: Effective (Eff) following Effective (EffEff-Eff); ineffective (InEff) following effective (Eff-InEff); ineffective following ineffective (InEff-InEff ) and effective following ineffective ( InEff-Eff)). The presentation of S1 and S2 are indicated in each panel by dashed vertical lines, with 0 corresponding to onset of S1. Color scales were equated across drug conditions.


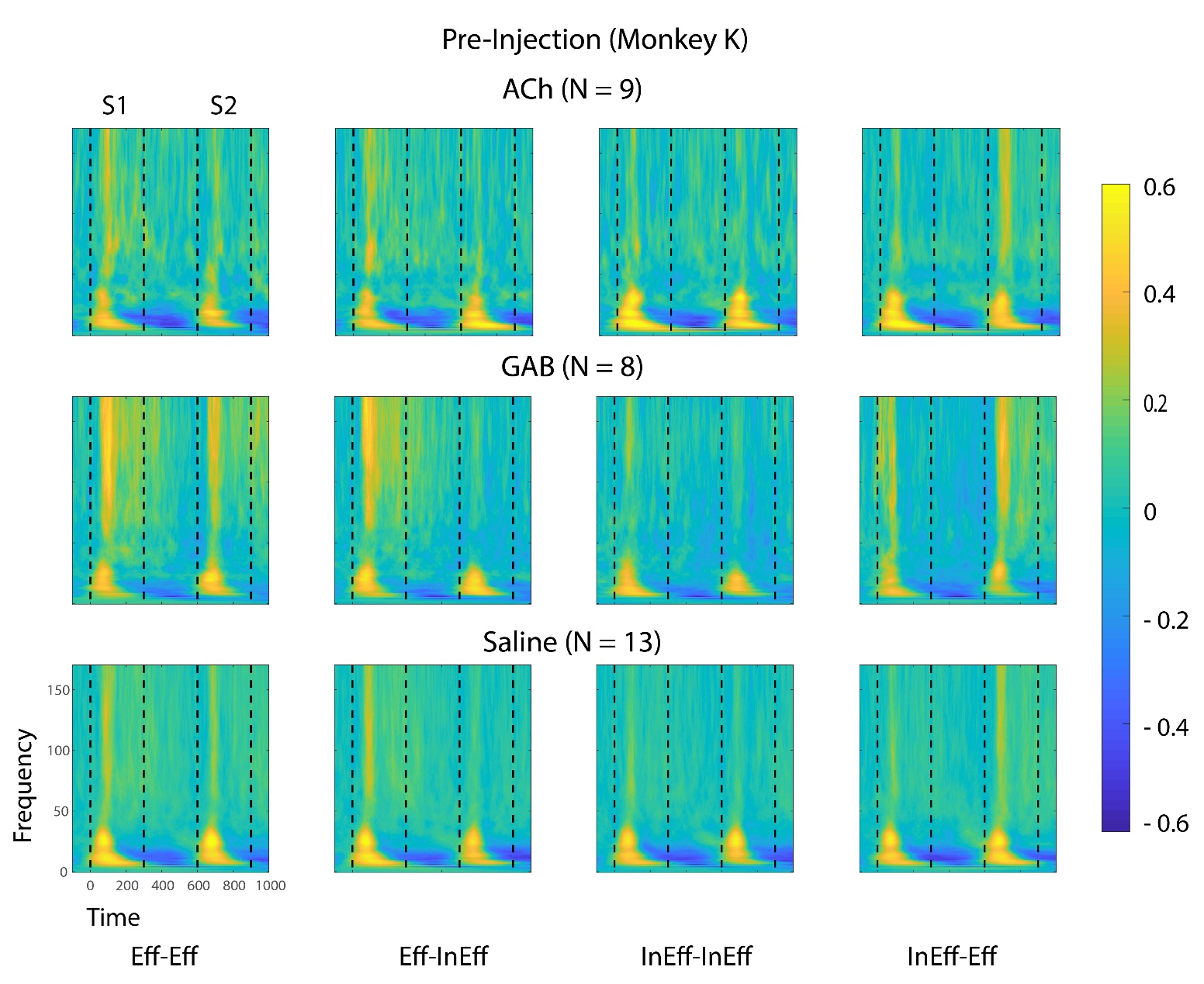


**Figure S3. Time-frequency LFP power plots before drug injections in monkey K**. Same conventions as in Fig S2.


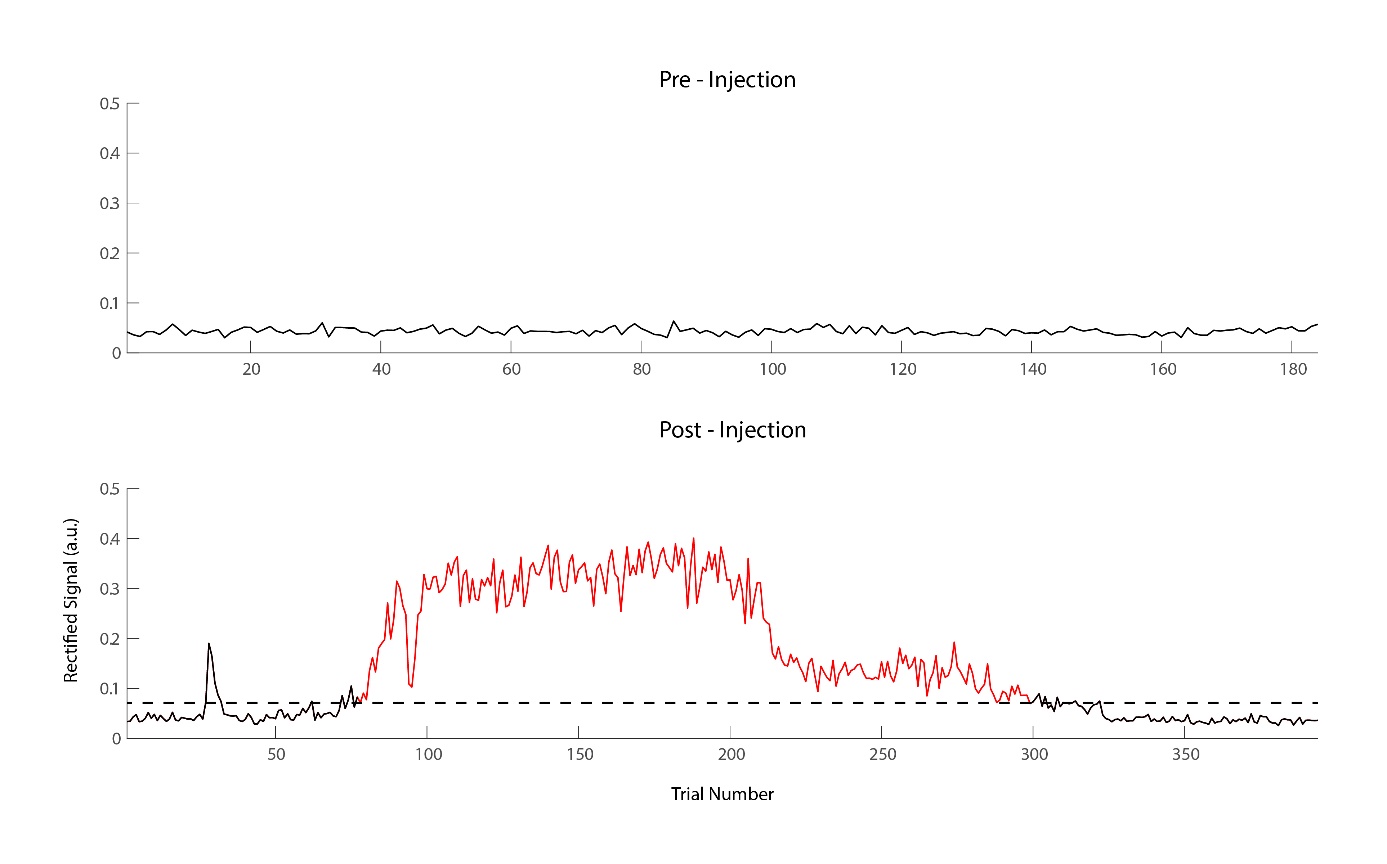


**Figure S4. Illustration of the procedure to define the post-injection analysis period for the ACh drug condition.** The rectified broadband neural signal is plotted as a function of trial number before (upper panel) and after (bottom panel) injection for one example recording session. The onset of the post-injection analysis period (red in bottom panel) was defined as the time at which the mean neural signal strength, operationalized as the rectified broadband signal, was greater in 10 consecutive trials than the 90th percentile of the signal strength (threshold, indicated by the horizontal line in bottom panel) in the pre-injection trials. Similarly, the end of the analysis period was defined by a trial above the same threshold that was preceded by 9 consecutive trials above the threshold. Note that this analysis period was defined without identifying the stimulus conditions (e.g. S1 versus S2) or the stimulus sequences and thus could not have biased an effect of ACh on adaptation.
